# Factors Associated with Self-Reported Repeat HIV Testing after a Negative Result in Durban, South Africa

**DOI:** 10.1371/journal.pone.0062362

**Published:** 2013-04-23

**Authors:** Susan Regan, Elena Losina, Senica Chetty, Janet Giddy, Rochelle P. Walensky, Douglas Ross, Helga Holst, Jeffrey N. Katz, Kenneth A. Freedberg, Ingrid V. Bassett

**Affiliations:** 1 Division of General Medicine, Massachusetts General Hospital, Boston, Massachusetts, United States of America; 2 Division of Rheumatology, Department of Medicine, and Department of Orthopedic Surgery, Brigham and Women’s Hospital, Boston, Massachusetts, United States of America; 3 Department of Biostatistics, Boston University School of Public Health, Boston, Massachusetts, United States of America; 4 McCord Hospital, Durban, South Africa; 5 Division of Infectious Disease, Massachusetts General Hospital, Boston, Massachusetts, United States of America; 6 Division of Infectious Disease, Brigham and Women’s Hospital, Boston, Massachusetts, United States of America; 7 St. Mary’s Hospital, Mariannhill, Durban, South Africa; 8 Harvard University Center for AIDS Research (CFAR), Boston, Massachusetts, United States of America; 9 Department of Epidemiology, Harvard School of Public Health, Boston, Massachusetts, United States of America; 10 Medical Practice Evaluation Center, Department of Medicine, Massachusetts General Hospital, Boston, Massachusetts, United States of America; Public Health Agency of Barcelona, Spain

## Abstract

**Background:**

Routine screening for HIV infection leads to early detection and treatment. We examined patient characteristics associated with repeated screening in a high prevalence country.

**Methods:**

We analyzed data from a cohort of 5,229 adults presenting for rapid HIV testing in the outpatient departments of 2 South African hospitals from November 2006 to August 2010. Patients were eligible if they were ≥18 years, reported no previous diagnosis with HIV infection, and not pregnant. Before testing, participants completed a questionnaire including gender, age, HIV testing history, health status, and knowledge about HIV and acquaintances with HIV. Enrollment HIV test results and CD4 counts were abstracted from the medical record. We present prevalence of HIV infection and median CD4 counts by HIV testing history (first-time vs. repeat). We estimated adjusted relative risks (ARR’s) for repeat testing by demographics, health status, and knowledge of HIV and others with HIV in a generalized linear model.

**Results:**

Of 4,877 participants with HIV test results available, 26% (N = 1258) were repeat testers. Repeat testers were less likely than first-time testers to be HIV-infected (34% vs. 54%, p<0.001). Median CD4 count was higher among repeat than first-time testers (201/uL vs. 147/uL, p<0.001). Among those HIV negative at enrollment (N = 2,499), repeat testing was more common among those with family or friends living with HIV (ARR 1.50, 95% CI: 1.33–1.68), women (ARR: 1.24, 95% CI: 1.11–1.40), and those self-reporting very good health (ARR: 1.28, 95% CI: 1.12–1.45).

**Conclusions:**

In this high prevalence setting, repeat testing was common among those undergoing HIV screening, and was associated with female sex, lower prevalence of HIV infection, and higher CD4 counts at diagnosis.

## Introduction

Routine screening for HIV infection can lead to earlier detection and treatment compared to screening only those experiencing symptoms of infection [1]. Repeated screening for HIV has been linked to higher CD4 cell counts when HIV infection is diagnosed which, if followed by prompt treatment with antiretroviral therapy, in turn reduces mortality [2], [3]. Because treatment lowers viral load and infectivity, a further benefit of early detection is reduced transmission of the virus [4].

WHO guidelines recommend routine screening for HIV in high prevalence areas.

In South Africa, national prevalence was 17% among those 15 to 49 years old in 2008, but was as high as to 26% in KwaZulu Natal (KZN) Province [5]. Young women are the most affected group, with prevalence of 33% among 25 to 29 year old females [5]. The South African National Strategic Plan (2007) [6] had ambitious goals including screening 25% of the population each year. The result has been a marked increase in HIV testing, especially among women. In 2008, 57% of women and 43% of men had ever been tested and 29% of women and 20% of men had been tested in the prior 12 months [7]. Higher rates of testing have been found among women of childbearing age and those with young children [8].

There are many barriers to HIV testing, including concerns about privacy and stigmatization [9], [10], lack of understanding of how HIV is transmitted, fear that one is infected [11], [12] or belief that one is not at risk for infection [12], [13], [14] and lack of knowledge about or access to treatment [15], [16], [17]. Acceptance of repeat testing further requires acknowledging that one remains at risk for future infection even after having received a reassuring negative result. Indeed, having been tested previously is a common reason for refusing a subsequent HIV test [13], [14], [18].

We conducted an observational study of patients presenting for HIV testing at the outpatient departments of two hospitals in KZN to assess HIV prevalence, assessing characteristics associated with repeat testing in this high prevalence setting. We compared those testing for the first time with those reporting having previously tested negative for HIV (repeat testers) to examine factors associated with repeat testing. We considered factors shown to predict HIV prevalence or first-time testing rates: demographics, knowledge of HIV [19] and friends or family living with HIV [20], and the circumstances of testing (provider-initiated, opt-out) [1]. We hypothesized that repeat testing would be more common among those who were younger and female, since pregnancy is a common occasion for HIV screening.

## Methods

### Setting and Participants

We analyzed data from the South African Test, Identify and Link (STIAL) Cohort which has been described in detail elsewhere [21], [22]. The cohort comprises adults who presented for HIV testing at the outpatient departments of two South African hospitals between November 2006 and August 2010. The participating hospitals were McCord Hospital, located in Durban (the urban site), and St Mary’s Hospital Mariannhill, in a largely rural area outside Durban (the rural site). Patients paid a fee for care in the outpatient department of 140–200 ZAR (US$18-26) at McCord Hospital and 50–70 ZAR (US$6-9 2008) at St Mary’s Hospital. Both hospitals changed from testing by physician referral to an opt-out HIV testing policy approximately one year into the study period. Under opt-out testing, dedicated counselors placed in the outpatient departments offered patients tests prior to their clinician visit. Both before and after the opt-out policy was adopted, patients were able to self-present for testing, independent of a clinician referral.

Patients were eligible for enrollment if, based on self-report, they had not been found to be HIV-infected in any previous test, were 18 years of age or older, Zulu or English speakers, not pregnant, were willing to share their HIV test results with study staff, and had no cognitive limitations that precluded giving informed consent. Patients who were confined to a stretcher or wheelchair were excluded to prevent study participation from interfering with their need for immediate care.

### Ethics Statement

The study was approved by the Ethics Committees of McCord Hospital and St. Mary’s Hospital Mariannhill, and the Partners HealthCare Human Research Committee in Boston, MA, USA. All participants provided written, informed consent.

### Screening Protocol

Patients were approached by study staff while they were waiting to undergo rapid HIV testing. Before the test and after giving informed consent, participants completed an orally administered questionnaire in Zulu or English. The questionnaire included:

- *demographics*: gender, age, and parental status (with or without children)
- *current health status*: self-assessed as very good, good, fair, poor, or bad
- *reason for having the HIV test that day*: referred by a healthcare provider vs. self-initiated testing
- *awareness of others living with HIV*: awareness of family, friends, or co-habitants infected with HIV
- *knowledge about HIV*: agreement or disagreement with a series of 4 statements about HIV. A knowledge score was calculated as the number of items correct (range: 0–4). The statements were: “There are medicines available to help people with HIV live longer”; “A person with HIV can look and feel healthy”; “There is a vaccine/medicine that can stop people from getting HIV”; and “All women who are HIV positive will have babies born with HIV”.
- *prior HIV testing history*: self-report of ever testing for HIV before anywhere, and if so, the number of times previously tested.

Participants usually received their HIV test results within 30 minutes. Those found to be HIV-infected were referred to the HIV clinic at the site and offered CD4 testing that day, with results available within 2 weeks. Medical record review was conducted to obtain HIV test results and CD4 counts performed within 12 weeks of enrollment.

### Statistical Analyses

Participants for whom a conclusive HIV test result was not available were excluded from analyses. We present prevalence of HIV infection by prior testing status at enrollment and median CD4 counts for those who were HIV-infected. Difference in prior testing rates was assessed using chi-squared tests. Differences in median CD4 counts were tested using a Wilcoxon rank-sum test.

We obtained the HIV status at enrollment from the medical record but relied on participant self-report for history of prior testing and test results. In analyses of factors associated with testing after a prior negative result, we excluded those who tested HIV-infected at enrollment because, among this group, we cannot be certain that all prior HIV test results were negative. Among participants HIV negative at enrollment, we know that the results of all prior tests were negative and therefore the enrollment test represents a test after a prior negative result.

We assessed differences in participant characteristics between those who did and did not report having had a prior HIV test using chi-squared tests, except for knowledge score which was tested using a Wilcoxon rank-sum test. Participant age was dichotomized at the median (<35 vs. > = 35 years). We dichotomized self-assessed health status as very good vs. all other. Linear trends in percent reporting prior testing by health self-ratings were assessed with logistic regression.

We estimated relative risks (RR’s) and 95% confidence intervals (CI) for prior testing by participant characteristics using a generalized linear model with a poisson distribution, a log link function and robust standard errors. We also present adjusted relative risks (ARR’s) and CI’s for these characteristics from a model predicting prior testing that includes participant gender, age group, reason for HIV testing, awareness of friends or family members with HIV infection, and knowledge of HIV and enrollment site (rural vs. urban).

All analyses were performed using Stata statistical software (StataCorp, 2008. Stata Statistical Software: Release 10. College Station, TX: Stata Corporation).

## Results

### Enrollment and Testing

**Figure 1:**
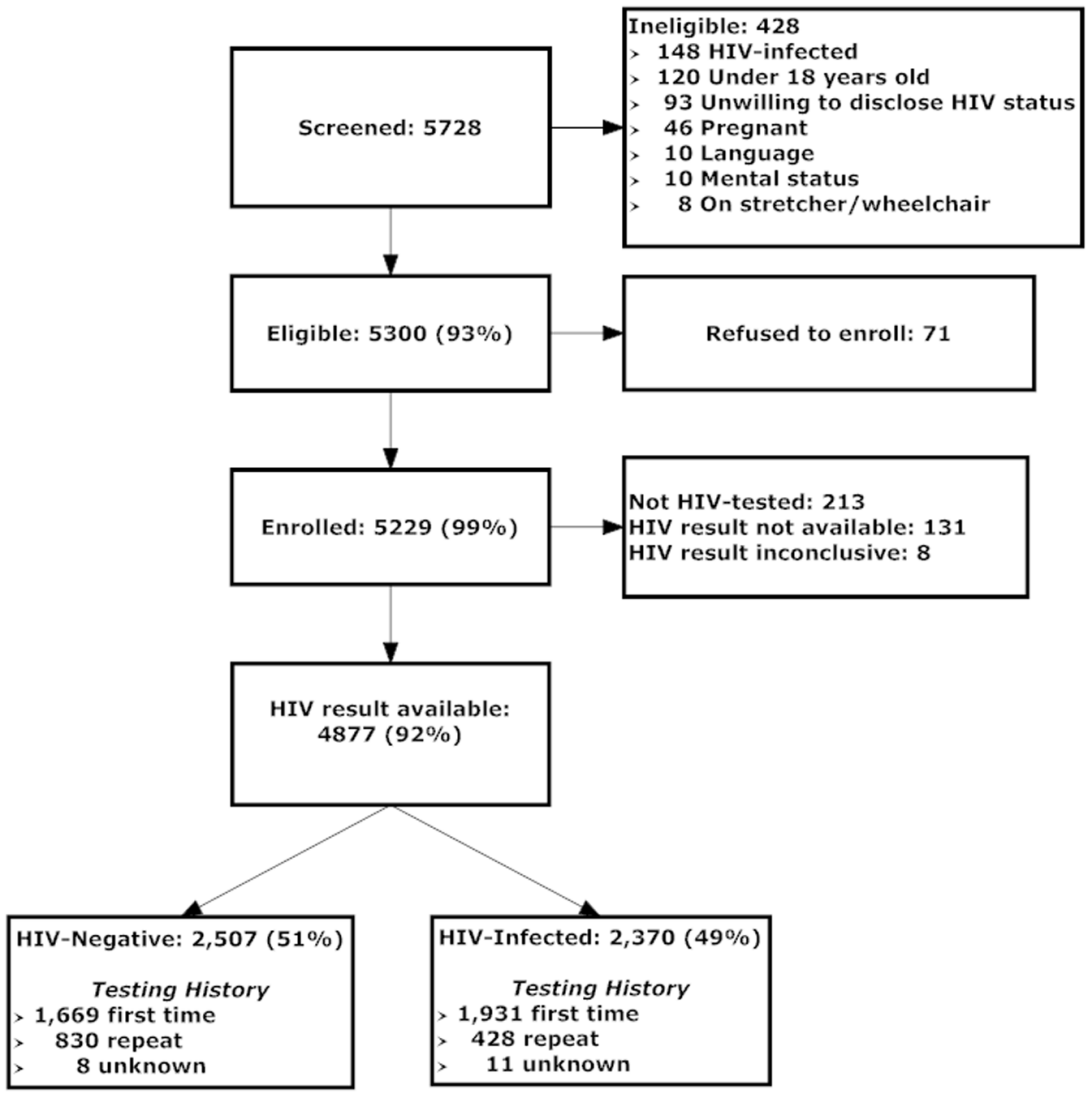
Cohort Enrollment and Testing.

Between November 2006 and August 2010, 5,728 patients were screened, of whom 93% (5,300/5,728) were eligible (Figure 1). Nearly all (99%, 5,229/5,300) of those eligible enrolled. Conclusive enrollment HIV test results were available for 4,877 participants (93%, 4,877/5,229), of whom approximately half (49%, 2,370/4,877) were HIV-infected. Overall, a quarter of participants reported having been tested for HIV prior to enrollment (26%, 1,258/4,877, Table 1). Compared to those having their first HIV test, repeat testers were less likely to be HIV-infected at enrollment (34% vs. 54%, p<0.001).

**Table 1:** HIV status and CD4 count among those HIV-infected at enrollment by HIV testing history.

HIV Testing History	Total		HIV Status				CD4 Count		
			Not Infected		Infected		N	Median	IQR*
	N	%	N	%	N	%			
First-time tester	3,600	74	1,669	46	1,931	54	1,183	147	58–286
Repeat tester	1,258	26	830	66	428	34	246	201	90–366
1 previous test	765	16	436	57	329	43	199	198	87–360
2 or more previous tests	493	10	394	80	99	20	47	235	128–514
Unknown testing history	19	<1	8	42	11	58	7	159	51–342
Total	4,877	100	2,507	51	2,370	49	1,436	156	62–298

* IQR: interquartile range.

### Repeat Testing and CD4 Counts

CD4 counts were available for 61% (1,436/2,370) of those found HIV-infected. Median CD4 count was higher among those reporting a previous negative HIV test (201/µl, IQR: 90–366 cells/µl) than those reporting no previous test (147/µl, IQR: 58–286/µl, p<0.001). Participants with two or more previous tests had a higher median CD4 count than those with only one previous test, but this difference was not statistically significant (235/µl, IQR: 128–514, vs. 198/µl, IQR: 87–360, p = 0.12).

### Factors Associated with Prior Testing

Of those participants who were HIV-negative at enrollment, approximately half were women, less than 35 years old, self-referred for testing and enrolled at the rural site (Table 2). The mean HIV knowledge score was 3.3, indicating that participants answered more than 3 of 4 questions correctly on average. Only 20% reported being aware that someone they knew was HIV-infected.

**Table 2:** Characteristics of participants HIV negative at enrollment.

Characteristic	Total	Tested prior to enrollment			RR* (95% CI)	ARR** (95% CI)
		No	Yes	P***		
	N (%)	N (%)	N (%)			
Female	1,400 (56)	887 (53)	513 (62)	<0.001	1.27 (1.13–1.43)	1.24 (1.11–1.40)
Age <35 years	1,114 (45)	696 (42)	418 (51)	<0.001	1.28 (1.14–1.43)	1.10 (0.98–1.23)
Very good health	395 (16)	182 (11)	213 (26)	<0.001	1.84 (1.64–2.06)	1.28 (1.12–1.45)
Self-referred for enrollment testing	1,117 (45)	633 (38)	484 (59)	<0.001	1.74 (1.55–1.95)	1.28 (1.14–1.43)
Family/friend with HIV	495 (20)	205 (12)	290 (35)	<0.001	2.18 (1.97–2.42)	1.50 (1.33–1.68)
HIV knowledge score (mean, SD)	3.3 (1.01)	3.3 (1.08)	3.4 (0.83)	<0.001	1.12 (1.05–1.19)	1.10 (1.03–1.18)
Rural site	1,099 (44)	558 (33)	541 (65)	<0.001	2.38 (2.12–2.69)	1.98 (1.72–2.28)
Tested under opt-out policy	1,970 (79)	1,236 (74)	734 (88)	<0.001	2.05 (1.70–2.48)	1.51 (1.25–1.83)
Total	2,499	1,669 (67)	830 (33)			

* RR: unadjusted relative risk for prior testing.

** ARR: adjusted relative risk for prior testing, adjusted for all characteristics shown.

*** P for bivariate comparison.

In bivariate analyses, participants with prior testing differed significantly from those with no prior testing on all characteristics examined, with those reporting prior testing having a higher mean HIV knowledge score and being more likely to be women, to be under 35 years old, to know someone with HIV, to have referred themselves for HIV testing, to report being in very good health, to have enrolled at the rural hospital and to have enrolled after the opt-out policy began.

A multivariate model of repeat testing was assessed including all factors in Table 2. Fifty-nine participants were missing a value for one of the factors and were excluded from the analysis. All factors remained statistically significant in the multivariate analysis except for age group. Being aware of a friend or family member with HIV was associated with a 50% higher rate of prior testing (ARR: 1.50, 95% CI: 1.33–1.68). Higher rates of prior testing were also associated with being female (ARR: 1.24 (1.11–1.40), self-referring for testing (ARR: 1.28, 95% CI: 1.14–1.43), reporting very good health (ARR: 1.28, 95% CI: 1.12–1.45) and having higher HIV knowledge scores (ARR: 1.10, 95% CI: 1.03–1.18).

**Figure 2:**
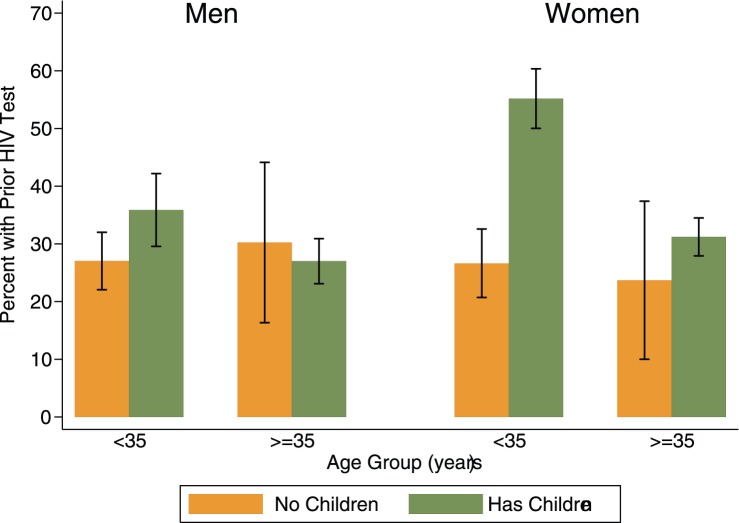
Percent with prior HIV test among those HIV negative at enrollment, by gender, age group, and parental status.

Women were more likely than men to report a prior HIV test (37% vs. 29%, p<.001), but the gender difference was confined to younger women. Figure 2 depicts prior testing rates by gender, age group and parental status (any children vs. no children). The highest rate of prior testing (55%) was observed among women under 35 years old with children, while rates did not differ significantly among the other groups.

**Figure 3:**
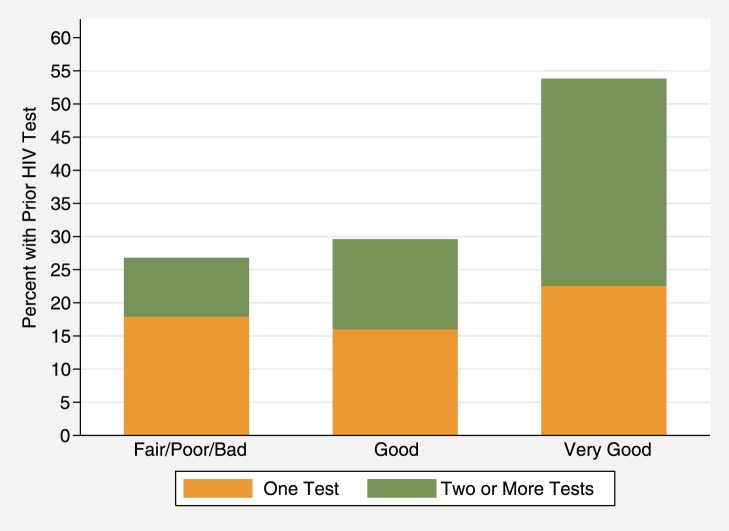
Percent with prior HIV test among those HIV negative at enrollment, by self-reported health status (linear trend p<.001).

The rate of reporting a history of any prior HIV tests rose with increasing self-assessed health status from 27% among those reporting the poorest health to 54% among those describing their health as very good, (p<0.001, Figure 3). Similarly, the rate of reporting two or more prior tests increased with health self-rating, from 9% of those with poor health to 31% of those with very good health (p<0.001).

## Discussion

In this observational study of a large cohort of outpatients seeking HIV testing in Durban, South Africa, we found a very high prevalence of HIV infection. Twenty-six percent of those enrolled were repeat testers, and repeat testing was more common in those who were not infected at enrollment (33% compared to 18% for those HIV-infected). Among those HIV-infected at enrollment, CD4 cell counts were quite low, but significantly higher in repeat testers (201 vs. 147/µl). We found repeat testing was associated with demographic characteristics, specifically younger age and female gender, and with knowing someone living with HIV. Repeat testing was less common among those referred by healthcare workers compared to self-referred participants yet more common when the clinic had a routine, opt-out testing policy. Prior testing and the number of prior tests were positively associated with self-rated health status.

Cross-sectional community-based surveys indicate that a substantial proportion of the population in sub-Saharan Africa have been tested for HIV at least once. Nearly half of those reached in a population-based survey in Botswana [12] reported having had an HIV test. In a rural community in South Africa, rates varied with gender, with 39% of women but only 17% of men surveyed reporting have been tested [8]. Repeat testers have been observed in relatively high proportions among those presenting for testing in some settings. A population-based study conducted in a peri-urban community in SA found 71% of those who responded to a request to report for HIV testing had been tested before [23]. At a mobile clinic in Capetown, SA, 58% of men being screened were repeat testers [24], and a similar rate was seen among all testers at a rural Zambian clinic [25]. In contrast, in an earlier study of our urban hospital, the rate was 35% [17] and the present study found only a quarter of participants were repeat testers. It may be that local factors like testing venue, attitudes [26] and background HIV prevalence [27] influence the observed rates of repeat testing.

Delayed diagnosis of HIV infection can have a serious negative impact on treatment outcomes. Patients with CD4 counts under 200 at diagnosis have much higher mortality rates [3] and are more likely to be diagnosed with an AIDS-defining illness [28]. Frequent, repeated HIV screening can shorten the period from infection to diagnosis, as seen in our HIV-infected repeat testers who had higher CD4 counts than the first time testers, and offers the hope of preventing disease progression and mortality [2].

Opt-out or provider-initiated testing policies have been implemented to promote HIV testing in high prevalence settings. These programs incorporate testing into routine visits, thereby reducing both logistical barriers and destigmatizing HIV screening. Such efforts have generally resulted in markedly greater uptake of HIV testing, although to varying degrees [17], [29], and may yield more late-stage diagnoses compared to voluntary counseling and testing [30]. Because opt-out testing policies target patients who are already connected to health services, they may be especially likely to reach those who have already been tested for HIV and promote repeat testing. Patients who have come to view HIV screening as routine care may be comfortable requesting screening in settings lacking an opt-out policy. We found the highest rate of repeat testing among younger women with children, a group whose HIV screening rates have been boosted by provider-initiated prenatal screening programs [8]. The women in our study were not pregnant at the time they presented for the enrollment test which suggests that the increasingly common experience of testing during pregnancy may promote acceptance of the notion of HIV screening as routine healthcare.

Basic knowledge about HIV transmission and treatment was high among our participants, with many correctly answering all the HIV knowledge items. Only 20% reported that they knew someone living with HIV, a remarkably low figure given the size and duration of the HIV epidemic in KZN, suggesting disclosure of HIV status is not common. This is consistent with a recent qualitative study in KZN which found reluctance to screen for HIV because of fear of discrimination, even within a community that is well-informed about HIV [16]. Education about HIV is not sufficient to eliminate the social costs of learning one is infected. Repeat testing was more common among those who acknowledged knowing someone living with HIV. This may be the result of perceived vulnerability to HIV infection because of a personal connection to an infected person. It might also indicate a relative normalization of HIV screening due to social proximity to someone who has disclosed being HIV-infected.

It is notable that any prior testing and multiple prior tests were associated with greater feelings of well-being. This may in part reflect that people feel healthy when they have had one or more negative HIV test results, but it also shows a continued willingness to test despite feeling healthy and implies an understanding that a person who feels well can be HIV-infected. Screening people who feel well is one key to earlier diagnosis of infection.

This study has a number of limitations. It is an observational study in which we rely on self-report for participants’ testing history. It is possible that participants who indicated that they were first-time testers had actually previously tested positive for HIV but were unwilling to disclose that information, which would falsely increase the difference in prevalence between first-time and repeat testers. CD4 counts were unavailable for 40% of those who were HIV-infected. During the study period, both of the hospitals implemented an opt-out HIV testing policy. We include this factor in our multivariate model to attempt to control for it, but testing policy is confounded with time, and it is possible that some of the increase in repeat testing associated with the opt-out policy is due to temporal trends in testing. Finally, we were unable to examine the effect of proximity to a testing site on HIV testing behavior.

In summary, in two hospitals in KZN with a high prevalence of HIV infection, a quarter of those presenting for HIV testing had previously tested negative. Repeat testers found to be HIV-infected at enrollment had higher CD4 counts at detection. A history of prior testing was more common among young women, those who know someone living with HIV, and those with good self-rated health status. Repeat testing increased when opt-out testing was implemented. Routine screening is beneficial, resulting in earlier detection. Future research should be directed toward determining optimal retesting frequencies for high prevalence settings.
